# Sociocultural factors and perceptions associated with voluntary and permanent relocation of flood victims: A case study of Sekondi-Takoradi Metropolis in Ghana

**DOI:** 10.4102/jamba.v9i1.303

**Published:** 2017-04-24

**Authors:** Isaac Y. Addo, Samuel Y. Danso

**Affiliations:** 1 Department of Population and Health, University of Cape Coast, Ghana; 2 Department of Geography and Regional Planning, University of Cape Coast, Ghana

## Abstract

Flooding is a major problem in many developing urban centres in Ghana, including the Sekondi-Takoradi Metropolis (STM). Residents who are living close to the Anankwari, Kansawura and Whin rivers in the metropolis often experience flooding when the rivers overflow their banks, resulting in lives being lost, people being displaced and properties being destroyed. One durable solution to the flooding problem is voluntary and permanent relocation of ‘vulnerable’ residents; but this form of solution cannot be achieved without a clear understanding of the sociocultural factors that influence the decision-making process. This study uniquely investigated the sociocultural and economic factors affecting voluntary and permanent relocation of flood victims, using Eshiem, Kansawurodo and Whindo communities as a case study. Employing a mixed cross-sectional design method, 207 heads of households were selected to fill in questionnaires; interviews were conducted with nine representatives of the traditional councils, and areas affected by flooding were photographed. The findings show that voluntary and permanent relocation was overlooked by most flood victims due to perceived inability to rent new places owing to low incomes, fear of losing income-generating ventures that serve as sources of livelihoods, hope of gaining income from the oil production within the region and the need for restitution from government before evacuation. From a sociocultural viewpoint, they felt uncomfortable with losing ancestral lands and landed properties as well as breaking long-standing ties with their community folks and other networks. Flood victims’ willingness to stay in the flood-prone communities was also influenced by duration of stay in the communities and ownership of landed assets. When considering voluntary and permanent relocation of flood victims as a durable solution in the future, these sociocultural and economic factors need to be carefully considered.

## Introduction

Over the years, flooding has caused devastating effects in many African countries, with major towns and cities in Kenya, Mozambique, Nigeria, Sierra Leone, Uganda, Zambia and Ghana still experiencing cases of flood disasters (Fukami & Herath 2009; Mendel 2006; Patt & Schroter 2008). In Ghana, numerous studies have been documented on the flooding problem, with the majority focusing on cases within the main capital city, Accra (Aboagye 2012; Afeku 2005; Amoako & Frimpong Boamah 2014; Appeaning & Adeyemi 2013; Asumadu-Sarkodie, Owusu & Jayaweera 2015; Ghana News Agency 2012; Karley 2009; Mumuni 2013; Rain et al. 2011). However, one major area that suffers from recurrent flooding is the Sekondi-Takoradi Metropolis (STM) in the Western region of Ghana; with Eshiem, Kansawurodo and Whindo communities identified as major flood-prone areas because of their proximity to the Anankwari, Kansawura and Whin rivers (Mendel 2006; Sekondi-Takoradi Metropolitan Assembly 2013). Drawing on a recently published newspaper article, Danquah (2016) reported that the metropolis experienced severe flooding on Thursday, April 07, 2016, resulting in the displacement of people, as well as the destruction of homes and other properties. On Sunday 4th May, 2013, a 13-year-old boy allegedly died in the metropolis due to flooding (Graphic Online 2014). In 2011 and 2009, more than a thousand people became homeless within the STM and valuable properties were destroyed after heavy rains caused the rivers to overflow their banks to neighbouring communities (Aklorbortu 2011; The International Federation’s Disaster Relief Emergency Fund 2009). As far back as 1971, heavy rainfall led to the collapse of about hundred dwelling houses within the STM, rendering thousands of people homeless (Daily Graphic 2015). In addition to the negative effects of the flooding incidents on human lives and properties, many developmental projects are delayed within the metropolis, as the government through the National Disaster Management Organisation (NADMO) and other state agencies channels resources to provide items such as food, clothing, shelter and mosquito nets to victims, which could have been used for other developmental needs (Sekondi-Takoradi Metropolitan Assembly 2013). In terms of economic cost, there is limited data on the exact flood-related expenditure in the STM. However, using national estimates as a guide, it has been documented that from 1900 to 2014, economic damages resulting from flooding in Ghana amount to about $780 500 000 (Asumadu-Sarkodie et al. 2015). Although these figures seem alarming, it is likely that the actual cumulative costs resulting from flooding are underestimated because of the difficulty in measuring costs of damaged items and financial losses during the recuperating periods (Okyere, Yacouba & Gilgenbach 2013).

Given the devastating effects of flooding, it is important to develop appropriate measures to at least reduce the impact on human lives and economies. Ghana has a number of policies such as the National Water Policy, Sanitation Policy and the Blue Agenda, which were focused on reducing the effects of disasters, including flooding (Ministry of Environment Science Technology and Innovation 2013). However, these policies, in addition to other locally enacted laws and regulations, have not been implemented effectively because of various socio-political factors (Ministry of Environment Science Technology and Innovation 2013). Article 36 (9) of the constitution of Ghana states that:

the State shall take appropriate measures needed to protect and safeguard the national environment for posterity; and shall seek cooperation with other states and bodies for purposes of protecting the wider environment for mankind. (Ministry of Environment Science Technology and Innovation 2013:10)

Here, the constitution clearly recognises the need to devise appropriate and urgent solutions to protect humanity and the environment (Danso & Addo 2016; Graphic Online 2014). These factors draw attention to the need to find durable solutions to the flooding incidents in the communities and beyond. Considering the case-peculiarity of communities close to the Anankwari, Kansawura and Whin rivers within the STM, a well-planned voluntary and permanent relocation of residents living along the river banks can be considered as a durable solution to the long-lasting effects of flooding which have existed in the metropolis as far back as 1971 (Daily Graphic 2015). Continuous stay at the same location makes the victims more vulnerable to future disasters (De Ville de Goyet, Marti & Osorio 2006). Similar recommendations have been given by Oteng-Ababio, Owusu and Addo (2011), and Ahadzie and Proverbs (2011) in their respective studies of different localities lying close to river bodies. It is known that permanent relocation as a durable solution to flood disasters is more likely to be effective when the idea is willingly initiated or received by the people at risk as well as victims of flooding (Leighton 2012). However, little is known about the sociocultural and economic factors that influence voluntary and permanent relocation of flood victims and populations at risk of flood disasters. This study therefore investigated the perceived sociocultural and economic factors influencing voluntary and permanent relocation of people vulnerable to flooding, using highly endangered communities (Anankwari, Kansawura and the Whin) in the STM as a case study. Understanding the perceptions of the people about voluntary and permanent relocation will assist in setting a clear platform for rethinking appropriate solutions to flooding problems in the country and similar regions.

## Study context, research design and methods

### Setting

**FIGURE 1:**
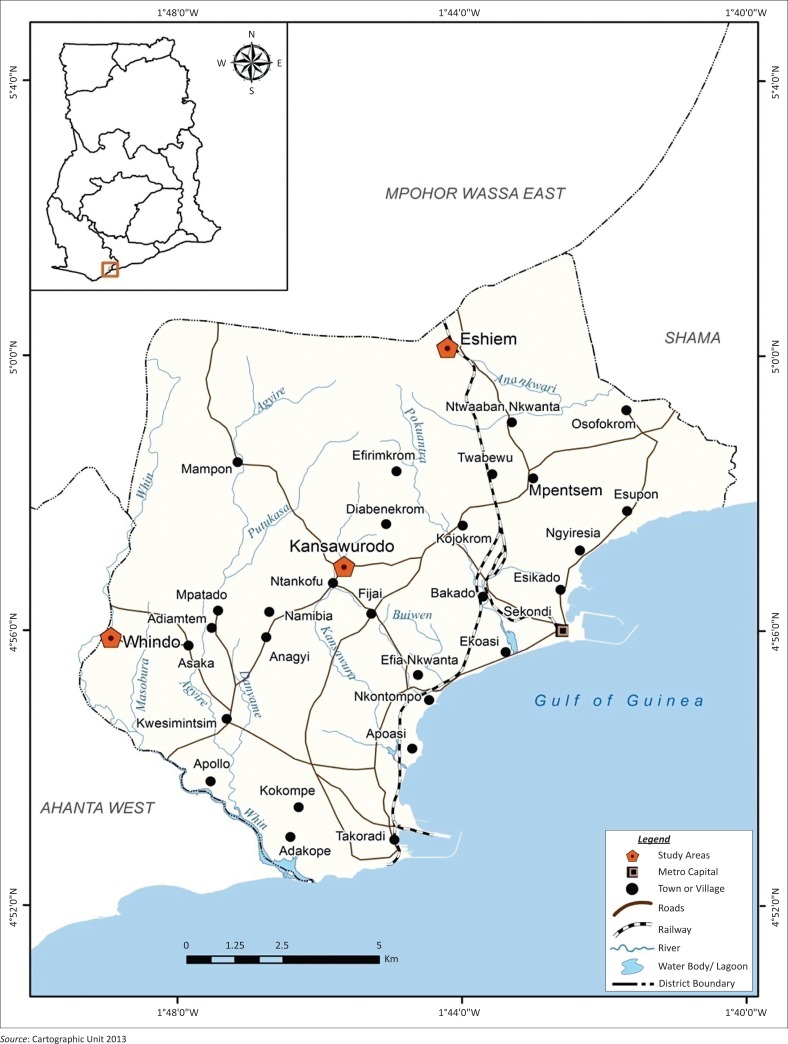
The Sekondi-Takoradi Metropolitan Assembly showing the study areas.

The STM in the Western region of Ghana was selected as the study area because residents experience recurring river flooding which makes them vulnerable to flooding effects, but many continue to live within the floodplains (Sekondi-Takoradi Metropolitan Assembly 2013). STM shares boundaries with Ahanta West District, Mpohor Wassa East, Shama District Assembly and the Gulf of Guinea (Figure 1). The total land area is about 49.78 km^2^. It is the third largest metropolis in the country with a population of 404 041 (Sekondi-Takoradi Metropolitan Assembly 2013). The metropolis is underlain by hard basement granites, gneisses and schists that are capped with faulted shale and sandstones. The ground water in the STM has a relatively high salt content because of its closeness to the sea. It has an undulating topography (Sekondi-Takoradi Metropolitan Assembly 2013). The low-lying areas within the region are interspersed with ridges and hills that range from 30 m a.s.l. to 60 m a.s.l. Kansawura, Anankwari and Whin are the major rivers which flow through the metropolis. The metropolis has moist tropical climate (Acheampong 2009). The annual mean temperature and rainfall are 22 °C and 2350 mm, respectively. The area has a double maxima rainfall. The major rainy season occurs between March and July, while the minor rainy season which is usually of short duration, but very intensive, takes place between September and November (Ghana Districts 2006). Eshiem, Kansawurodo and Whindo suburbs within the STM were purposively selected for the study because they are closest to the aforementioned major rivers, making them extremely risky and prone to flooding (Sekondi-Takoradi Metropolitan Assembly 2013). Detailed description of the STM has been reported elsewhere (Danso & Addo 2016).

### Design

A mixed, cross-sectional design method involving concurrent collection of quantitative and qualitative data was used in the study (Creswell 2003). All consenting household heads residing in the three most risky communities were considered for the study. Heads of households were selected because they are usually the final decision-makers in most flooding events and were assumed to be having rich knowledge about factors likely to affect voluntary and permanent relocation. Questionnaires were used to gather data from the heads of households; nine household heads who also double as community elders representing the traditional council of Eshiem, Kansawurodo, and Whindo were interviewed and certain areas within the communities were photographed. The community elders were interviewed based on the assumption that they have rich knowledge of the flooding issues within the community because of the responsibilities associated with their positions. The data covered the effects of flooding on flood victims, as well as the reasons why they continued to stay in such areas although their lives were at risks. The total populations of the communities (Eshiem, Kansawurodo and Whindo) were 1985, 4400 and 2107, respectively (Sekondi-Takoradi Metropolitan Assembly 2013).

### Procedure

From a frame of 1377 heads of households affected by flooding in all the communities, a sample size of 207 was scientifically determined. Systematic sampling technique was used in selecting the heads of households. This technique required the calculation of sample intervals for each community. This was done by dividing the number of households (h) of each community by the community’s sample size (z). Therefore, the sampling interval derived was seven for every community. At each community, the first household was randomly selected and the sampling interval was used to select the subsequent heads of households. At Whindo, for example, after the purposive selection of the first head of household, every seventh head of household was considered as a respondent. This procedure was repeated until the required sample assigned to each community was obtained. The sample size of each community was obtained by dividing the population of the households in a community by the entire household population (1377) of the three communities. The result was then multiplied by the sample size of 207 which is displayed in the following model:

$$\frac{\text{Number of households}\times 207}{\text{Total number of households in the three communities}}$$ [Eqn 1]

Therefore, a total number of 50, 100 and 57 heads of households were selected in Eshiem, Kansawurodo and Whindo, respectively. Three field assistants reading Masters Programmes at the University of Cape Coast in Ghana were trained to assist in the data collection. The questionnaire was pretested at Kwaprow in the Central Region of Ghana. The selection of Kwaprow was based on the fact that it shares some characteristics with the study area. For instance, it lies close to the Kakum River and also experiences intermittent flooding whenever it rains copiously. Permission was obtained from the Sekondi-Takoradi Metropolitan Assembly (STMA), and the chiefs and heads of households before the fieldwork. Introductory letter from the Department of Geography and Regional Planning was sent to the chiefs and gatekeepers before the commencement of the fieldwork.

### Analyses

After gathering the relevant data, the outcome was examined to check out mistakes, and the data were analysed using Statistical Product and Service Solution (version 16) software. Frequencies and percentages were used to analyse the numeric data as presented in tables and charts. The in-depth interviews were transcribed, and most recurring themes were manually selected and quoted to enrich the analyses.

## Results

### Background characteristics of respondents

Some background characteristics of the heads of households were generated. These were sex, attained level of education, average monthly income and the length of stay in the current community. As shown in Table 1, the distribution of respondents by sex indicates that males (53.1%) constituted the majority. This finding is particularly important because in many traditional Ghanaian homes, men often take the final decisions to migrate when disaster occurs (Daplah 2013). Table 1 further shows that the educational level of the respondents was quite low. About 49% had attained Junior High School education, followed by 17% who had attended Primary School, whereas 1% had attained Tertiary level of education. About half (50.2%) of the respondents reported that their monthly income was between 100 and 200 cedis (about $ 50), followed by 37% who earned below 100 cedis (about $25) per month (Table 1). It was also found that nearly three-quarters (74.4%) had lived in the community for 10 years or more (Table 1).

**TABLE 1:** Background characteristics of respondents.

Background characteristics	Frequency	Percent
**Sex**		
Males	110	53.1
Females	97	46.9
**Level of education attained**		
None	35	16.9
Primary	36	17.4
JHS	102	49.3
SHS	31	15.0
Tertiary	3	1.4
**Average monthly income (GH ¢)**		
Less than 100 ($25)	77	37.2
Between 100 and 200 ($25–50)	104	50.2
Between 200 and 300 ($50–75)	16	7.7
Above 300 (> $75)	10	4.8
**Length of stay in community**		
Less than a year	8	3.9
Between 1 and 4 years	22	10.6
Between 5 and 9 years	23	11.1
10 or more years	154	74.4

JHS, Junior High School; SHS, Senior High School; GH, Ghana.

$1 was equivalent to ¢4 at the time of the survey.

### Self-reported effects of flooding

**FIGURE 2:**
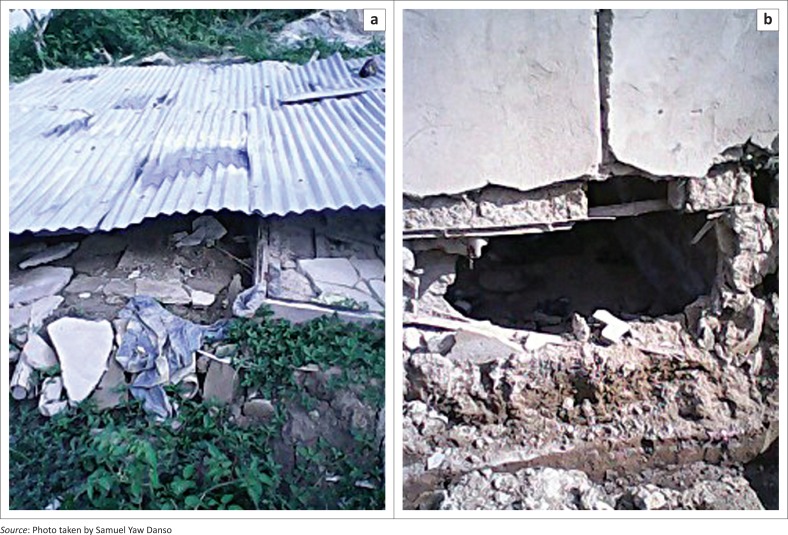
Destroyed buildings due to floods at Whindo. (a) Totally collapsed building due to flooding and (b) partially destroyed building due to flooding.

To set the discussion in the context of the study aim, it was necessary to firstly explore the effects of flooding on the lives of the residents. Multiple response scales were used to analyse the views of the flood victims, with respect to the impact of the flooding on their lives. As shown in Table 2, the responses could be broadly categorised into health, environmental, social, domestic and psychological effects. It was found that unusual malaria (63%) and cholera (28%) occurred during times of flooding, while severe injury and even deaths (32%) were also recorded. Figure 2 shows a location where one person died with three others injured during one flooding incident.

**TABLE 2:** Self-reported effects of flooding.

Self-reported effects of flooding	Frequency	Percent
**Health effects**		38.2
Household members get unusual malaria during floods	130	62.8
Outbreak of cholera or dysentery when floods occur	58	28.0
Flooding pollutes source of drinking water	61	29.5
Flooding	67	32.4
**Environmental effects**		72.7
Flooding brings filth, debris, dumped materials to the environment	166	80.2
There is bad stench after flooding events	135	65.2
**Effects on social responsibility**		66.2
Inability to go to work during floods	145	70.0
Inability of children to go to school during and after flooding	129	62.3
**Domestic effects**		55.8
Household properties such as furniture and electrical gadgets got destroyed due to flooding	161	77.8
House has developed cracks due to floods	70	33.8
**Psychological effects**		
Afraid whenever it begins to rain	166	80.2

**FIGURE 3:**
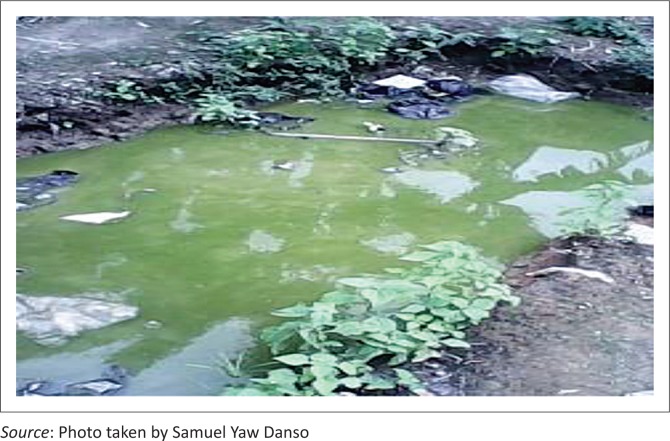
Stagnant water in a puddle at Kansawurodo after flood.

Figure 3 shows stagnant water resulting from heavy rains which became a breeding place for mosquitoes.

**FIGURE 4:**
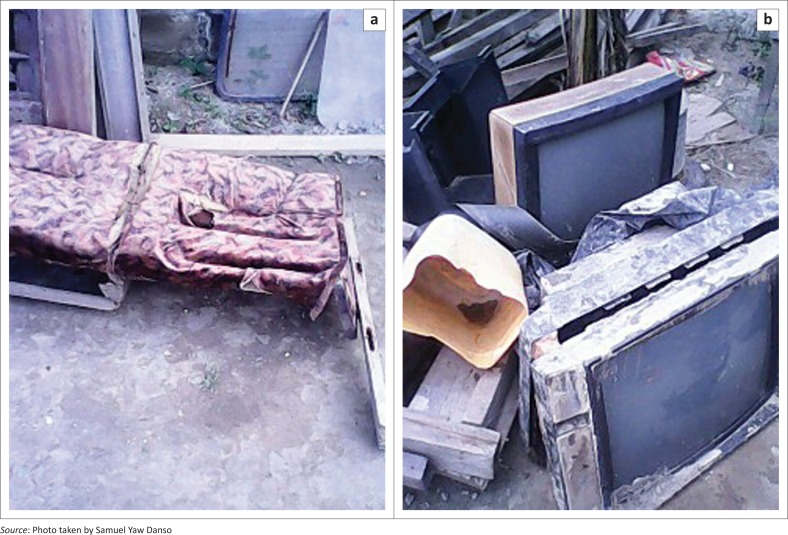
Some destroyed properties in a household at Eshiem. (a) Destroyed beds due to flooding and (b) destroyed television sets due to flooding.

Filth (80%) and bad odour (65%) were common during flooding, whereas students (63%) and workers (70%) could not attend to their usual duties. Household properties (78%), such as furniture and electrical gadgets, were significantly destroyed during flooding. Figure 4 confirms the lamentations made by some respondents.

One psychological effect of the flooding was the fear experienced by victims (80%) whenever it began to rain (Table 2). A 41-year-old woman at Eshiem lamented:

Look, the floods which occurred last year reached the level of my waist. Now, my heart beats very hard and fast when it rains, because what happened here last year (2012) was not a joke. (Female, Trader, 41 years)

**FIGURE 5:**
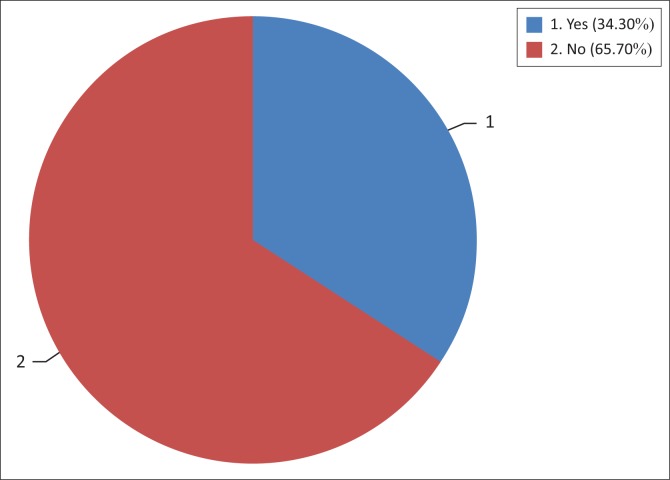
Respondents’ decision to relocate.

The study further sought to find out whether the flood victims would like to permanently relocate to safer areas. As indicated in Figure 5, about 66% did not opt for voluntary and permanent relocation, despite the associated dangers and discomforts associated with flooding.

### Perceptions of voluntary and permanent relocation as a durable solution to flood risk

**FIGURE 6:**
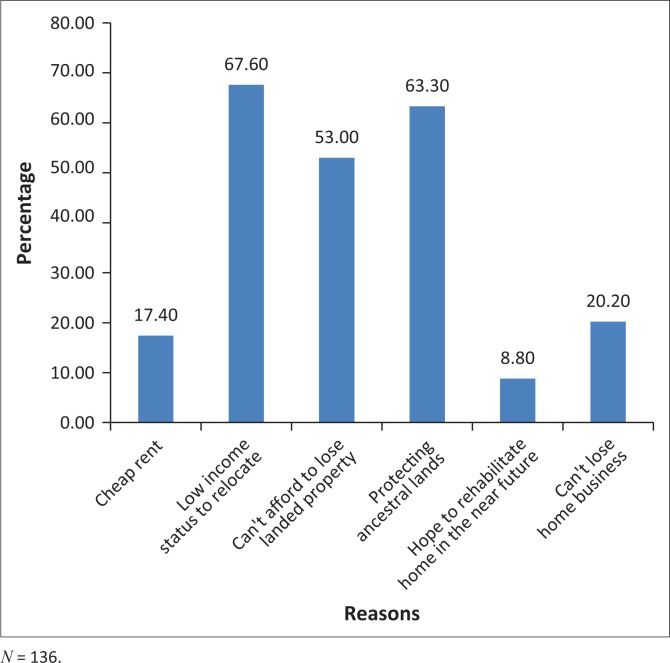
Self-reported reasons hampering relocation after flooding incidents.

Multiple response scales were used to analyse the rationale behind the flood victims’ unwillingness to permanently relocate to safer places. As shown in Figure 6, more than half of the flood victims opted to stay in the dangerous zones because of low incomes (68%), duty to protect ancestral lands (63%) and ownership of landed properties (53%).

In line with the quantitative results, emerging themes from the in-depth interviews show that majority of the community leaders were not willing to voluntarily leave the communities. The themes that emerged from the interviews can be broadly categorised as cultural beliefs, fear of losing source of livelihood, high cost of rent in STM because of the discovery of oil, fear of abandonment after relocation and unknown package for permanent relocation. A key informant narrated that they inherited the community lands from their forefathers and hence felt obligated to protect it. One community leader who had lived in the community for more than 40 years said:

This flooding problem wasn’t very big in about 20 years ago. I don’t think the problem is all about moving to a different area. Our grandfathers owned this land and we have also inherited it. That has been the custom before we were even born, and it will be disappointing to let go of our lands. I think that the STMA, NADMO and the government can help find other solutions to this problem. The main problem is the increasing population and dumping of refuse into the river bodies. (Male, Farmer, 48 years)

While the traditional owners of the lands considerably felt uneasy losing their lands and other landed properties, those without landed assets complained about cost of housing because of the discovery of oil in the STM and fear of losing their sources of livelihoods. A male head of household who doubles as a community leader and aged 52 years stated:

We have established petty businesses in this area. Moving to another place will be a big worry. You know … Housing has become a very serious problem since they discovered oil in the region. Migrants are moving into this place from other cities because of the oil; and cost of housing has shot up, all of a sudden. We are aware of the risk involved in living here, but we have no option. (Male, Trader, 52 years)

Additionally, some key informants drew attention to the point that voluntary and permanent relocation will be a disadvantage to them, unless they receive huge monetary packages in the form of compensation from government and humanitarian agencies such as NADMO.

A female respondent aged 47 years narrated:

Unless the government can give us attractive compensations to restart our businesses and pay rents, I don’t think moving from here (Kansawurodo) will be that easy. Some of us are not living here by choice. We are here because the system (economy) is hard. I think the possibility of relocation lies in the package given to us. (Female, Provision shop owner, 47 years)

## Ethical considerations

The study was allowed by the Department of Geography and Regional Planning of the University of Cape Coast in Ghana before the commencement of the data collection. Permission was obtained from the Sekondi-Takoradi Metropolitan Assembly, the chiefs and heads of households before the commencement of the fieldwork. Introductory letters from the Department were presented to the chiefs. All procedures followed were in accordance with the ethical standards of the University. Informed consent was obtained from all respondents. Research participants were informed about the purpose and objectives of the study before they gave their consents, and data have been kept confidential. Anonymity was ensured by not affiliating personal information of the respondents to the data.

## Discussion

The main purpose of this study was to explore the sociocultural and economic factors affecting voluntary and permanent relocation of flood victims living in flood-prone areas. To better understand the perceptions of the people about voluntary and permanent relocation, it was necessary to assess the extent to which the flooding events had affected their livelihoods. As demonstrated in the results, residents living in the Eshiem, Kansawurodo and Whindo communities had experienced various harsh effects from flooding. Broadly, the effects can be categorised as psychological, environmental, health, social and economic in nature. To a large extent, the psychological effect associated with the flooding events was intense, as more than three-quarters of the victims proclaimed that they were scared and apprehensive whenever it rained. Similar to this finding is the work of Tunstall et al. (2006) which pointed out that psychological effects were commonly reported after flooding, with anxiety mentioned as a usual problem.

Deposition of filth or debris on the land, and the destruction of the lands’ topography are some of the environmental effects resulting from the recurring flooding events. It was noted that sometimes the waste materials that were deposited on land during flooding were not cleaned entirely, leaving the environment filthy. Additionally, some flood victims mentioned that refuse were dumped into the rivers by some residents, which contributed to the flooding, when it rained heavily. Thus, the rivers in turn deposited the refuse on the banks after a severe rainfall. Deductively, the flood victims insinuated that human activities contributed to the flooding problem and its associated filth. In the various homes, properties were destroyed and buildings developed cracks. Buttressing this fact, personal observations showed that most buildings in the selected communities, especially at Eshiem, had been built with laterite without or with little cement, which made the buildings susceptible to the destructive force of the flood waters.

Economically, flooding incidents were associated with financial losses, especially when the destroyed properties were to be replaced. More than three-quarters of the respondents said that some of their household properties such as television sets, fridges, mattresses and sewing machines got destroyed during flooding. The community leaders also drew attention to the point that businesses were halted during flooding events, which depreciated their income levels and created ‘interim’ poverty, especially for those who had to borrow money and other resources to replace the destroyed properties. The flooding problem itself has created a ‘cycle of poverty’ and contributed to their inability to leave the dangerous areas, as affected people needed to replace destroyed properties, which in turn reduced their income levels and indirectly affected their ability to rent better places. Correspondingly, Aboagye (2012) in his study of the political ecology of environmental hazards in Ghana found that the prevalence of urban poverty has intensified the problem of human vulnerability to flooding. Thus, the temporary economic losses because of flooding is likely to increase people’s vulnerability to future flooding, as victims are likely to remain in the affected areas for long periods as a result of low-income levels.

Considering health effects resulting from the flooding, unusual outbreak of malaria was commonly reported by the flood victims. Cholera or dysentery was also reported by the flood victims, as echoed by the work of Mumuni (2013). Stench emanated from the flood waters which also compounded the plight and discomfort of the victims. This resulted from the spread of faecal matter in the neighbourhood, as a result of the collapse of liquid waste disposal systems during periods of flooding. The stagnant water found in places within the community after flooding served as breeding grounds for mosquitoes, which increased incidence of malaria. Some mentioned that their sources of drinking water were polluted, which, in part, was a contributory factor to the outbreak of cholera and dysentery. At worse, others died and suffered from injuries resulting from the flooding. However, the death toll was reportedly low and occasional because of the predictable nature of flooding events.

From a social perspective, more than half of the household heads complained that school children and workers were unable to go to school and work during flooding. As seen in the works of Ochola, Eitel and Olago (2010), flooding posed safety and hygiene threats to school children. This affected income generation and students’ performance as opined by the victims. Sometimes school materials such as books were destroyed during flooding. School buildings according to them served as evacuation centres that accommodated flood victims. Drawing on a study investigating the environmental hazards resulting from flooding in the Anloga, Dichemso, Aboabo and Amakom communities in Ghana, Oppong (2011) also found that flooding occurring in the communities resulted in life losses, destruction of properties, financial hardships and health problems. Similar findings were also highlighted by the Institute of Local Government Studies (ILGS) and International Water Management Institute (IWMI) (2012).

Looking at the various effects resulting from the flooding events, one would have thought that voluntary and permanent relocation would be embraced by the flood victims. However, findings from the study demonstrate that majority had lived in the area for a decade or more, and more than half of them opted to live in the area because of various sociocultural and economic reasons. From a sociocultural perspective, some residents who belonged to the traditional makeup of the communities highlighted the value they place on staying in an ancestral home, which to them, was a major reason why they would not opt for permanent relocation. To them, their forebears lived and were buried in these lands, and as a result, they cannot envisage leaving their locations for a reason which had existed for many years.

An elderly key informant said:

The elders say that no land hates the corpse (Proverb). But it is always an honour to keep what you have inherited from people who came before you. Leaving this place would mean that our traditional system may break down and there wouldn’t be any place to return and call as home. It is better to remove the waste sediments from the rivers and make them flow to prevent flooding when it rains. I think that is the main problem. Sometimes, the ‘gods’ become angry due to such behaviour and we all suffer. But leaving here wouldn’t be the ideal solution because our forefathers had survived it for many years. (Male, Fisherman, 62 years)

In their view, leaving the community life would mean a breakdown of the spiritual bond they have had with their ancestors (spirits of heroic family members who had died). After carefully analysing their responses, it was realised that the feeling of a spiritual bond between ancestors and the people was associated with residents who had lived in the community since birth or childhood. Another dimension of the sociocultural factor affecting decisions to voluntarily relocate was a feeling of strong community ties with social networks. In other words, some residents were not ready to lose close friends and relatives with whom they had formed strong ties since childhood. The implication drawn from this point was that the residents in the communities had a ‘we’ feeling, which made them regard themselves as a unit instead of individual families, and losing this form of lifestyle was problematic to a number of them. To some women who were household heads, they had formed various local associations and networks and leaving relatives and other close associates behind was quite difficult.

In addition to the cultural reasons for staying at the flood-prone communities, economic factors also influenced the option to stay in the communities. The discovery and production of oil in the region influenced decisions to stay in the flood-prone areas in three ways. First, residents who owned lands in the communities regarded land ownership and renting out of rooms as a form of lucrative business, as prices of rents increased as a result of large-scale migration of people to the metropolis for employment in the oil sector. Secondly, for residents who were paying rents, living in the flood-prone communities was relatively cheaper than living in bigger towns nearby. Given their small income levels, it was more economical to live in the communities for the mean time and relocate in the future when their income levels increased. Lastly, those engaged in businesses, petty trading and farming were not comfortable losing their customers, lands and sources of livelihoods. A male household head narrated:

Now, land and accommodation have become expensive after they discovered oil in this region. Even in our communities, cost of accommodation has risen and if you have land, it is an opportunity to make money, if you are able to build one or two structures (rooms). I can’t leave this opportunity when others want to move here to enjoy the opportunity we have now. Also, rents are more expensive in the neighbouring towns than in our communities and leaving to resettle in such places, is like creating your own inconvenience. (Male, Pensioner, 64 years)

Some feared facing economic hardships after they permanently relocate to an area where they would have to spend time to acclimatise and advertise their businesses. In part, some residents were interested in receiving monetary packages from government, which to them can influence their decisions to voluntarily relocate to restart life elsewhere. However, they felt the packages should be enough to enable them re-establish their businesses and also pay for their immediate cost of rents in a new location.

A male respondent in his late 40s said:

My brother, you know how the economy is rough. Some of us are living here not because we just want to be here, but because of monetary problems. I have four children and a wife, and they are all depending on my small income. Leaving this place will put me into severe economic hardship. I cannot afford to spend all my small income on rent. If the government can compensate us, that would help. But for now, things won’t be easy if we leave here. (Male, Factory worker, 48 years)

Some victims were unconvinced about voluntary and permanent relocation, as they felt they could be abandoned by government if they do not receive immediate help prior to permanent relocation. The finding supports Ahmed and Ahmed’s (1999) observation that most low-income residents in developing countries do not consider moving elsewhere after recession of floods.

To this end, the study findings indicate mixed reactions towards voluntary and permanent relocation of flood victims and populations at risk of flooding. Generally, responses from the victims proved that socio cultural and economic advantages derived from living in the flood-prone areas override the fear associated with the recurring flooding events. Similarly, Aboagye (2012) highlighted that many households in Accra lessen the impact of flooding by seeking to minimize its impacts while maximizing social and economic resources. However, reluctance to voluntarily and permanently relocate cannot be considered as an option for everyone in the communities, particularly for those who remained in the communities for economic reasons. Being born in the communities was a major setback to the acceptance of voluntary and permanent relocation, while migrants in the communities were more willing to permanently relocate, if their income levels increased.

## Limitations of the study

This study mainly contributes to knowledge by providing an understanding of sociocultural factors that affect voluntary and permanent relocation of flood victims in the context of recurring river flooding. For instance, the connection between oil discovery in the region and unwillingness to permanently relocate is a new contribution to the literature. Although the study contributes to understanding the sociocultural factors affecting decisions to voluntarily relocate after recurring flooding events, some limitations and challenges were encountered. Firstly, some heads of households were new residents of the community and had never experienced flooding incidents. In order to avoid ‘sham’ responses, the fieldworkers had to target households affected by flooding, which ‘slanted’ the original sampling frame. Secondly, the effects of the flooding were not uniform, as some experienced harsher conditions than others, which might have contributed to the variations in decisions to permanently relocate. Also, the study only focused on heads of households and did not capture the perceptions of other household members which would have enriched the study. Care should be taken when generalising the study findings to other areas (particularly towns and cities) with diverse cultural beliefs.

## Conclusion

It is conclusive from the study that the flooding incidents in Eshiem, Kansawurodo and Whindo communities had numerous effects on victims. Socio-economic activities, the environment and properties were heavily affected. Psychologically, flood victims were distressed or traumatised during flooding or whenever there were signs of rainfall. Homes were destroyed and people even died in severe cases. Despite the negative effects resulting from the recurring flooding, a significant proportion of the residents stayed in the dangerous zones because of various reasons. Decisions to stay in the communities were influenced by economic, cultural and social reasons such as low-income status, protection of cultural heritage, land ownerships, fear of losing source of livelihood and uneasiness to lose friends and relatives. However, the influence of these factors on decisions to stay in the communities varied by duration of stay in the communities and ownership of assets. It is therefore recommended that any consideration of voluntary and permanent relocation of flood victims in the future should consider the varying sociocultural and economic situations of the flood victims and populations at risk, in order to avoid post relocation distress and discomfort.
